# Antibacterial, Anticandidal, Phytochemical, and Biological Evaluations of Pellitory Plant

**DOI:** 10.1155/2020/6965306

**Published:** 2020-12-21

**Authors:** Mohammad Qadi, Nidal Jaradat, Saad Al-lahham, Iyad Ali, Murad N. Abualhasan, Naser Shraim, Fatima Hussein, Linda Issa, Ahmed Mousa, Abdalraziq Zarour, Amani Badrasawi, Aseel M. Baarah, Reem Al-Omari

**Affiliations:** ^1^Department of Biomedical Sciences, Faculty of Medicine and Health Sciences, An-Najah National University, Nablus, P.O. Box. 7, State of Palestine; ^2^Department of Pharmacy, Faculty of Medicine and Health Sciences, An-Najah National University, Nablus, P.O. Box. 7, State of Palestine

## Abstract

Pellitory plant (*Parietaria judaica* (PJ)) is one of the most widely used Arabian traditional medicinal plants due to its ability to cure several infectious diseases and other illnesses. The current study is aimed at assessing the phytoconstituents, antilipase, antiamylase, antimicrobial, and cytotoxic characters of the Pellitory plant (*Parietaria judaica* (PJ)). Phytochemical screening and procyanidin detection were conducted according to the standard phytochemical procedures. Porcine pancreatic lipase and *α*-amylase inhibitory activities were carried out using *p*-nitrophenyl butyrate and dinitrosalicylic acid assays, respectively. In addition, antimicrobial activity was determined utilizing a microdilution assay against several bacterial and fungal strains. Besides, the cytotoxic effect against HeLa cell line was tested employing 3-(4,5-dimethylthiazol-2-yl)-5-(3-carboxymethoxyphenyl)-2-(4-sulfophenyl)-2H-tetrazolium (MTS) assay. The quantitative test results revealed that the methanol fraction of PJ contains 18.55 ± 0.55 mg of procyanidin and has a potential *α*-amylase inhibitory activity compared with the antidiabetic drug Acarbose with IC_50_ values of 15.84 ± 2.25 and 28.18 ± 1.22 *μ*g/ml, respectively. Also, it has a potential antilipase activity compared to the commercial antiobesity drug, Orlistat, with IC_50_ values of 38.9 ± 0.29 and 12.3 ± 0.35 *μ*g/ml, respectively. The acetone, hexane, and methanol fractions have broad-spectrum antibacterial activity against the screened bacterial strains, while the acetone fraction has shown anticandidal activity with a MIC value of 0.195 mg/ml. The PJ hexane and acetone fractions decreased HeLa cell viability significantly (**p** value < 0.0001) by approximately 90% at the concentration of 0.625 mg/ml. The revealed outcomes showed that the methanol fraction has strong *α*-amylase and lipase inhibitory characters. Besides, acetone, hexane, and methanol fractions have broad-spectrum antibacterial activity, while the acetone fraction revealed potent antifungal activity against *Candida albicans*. Moreover, at low concentrations, hexane and acetone fractions have potent cytotoxic and antiproliferative activity against HeLa cancer cells. Nevertheless, PJ acetone, hexane, and methanol fractions can serve as an effective source of natural products to develop new antiobesity, antidiabetic, antimicrobial, and anticancer agents.

## 1. Introduction

Humans, since time immemorial, have relied on herbs and other natural products for the recovery and prophylaxis of many illnesses. In addition to their effective potentials in the therapeutic features, herbal products are easier to obtain, less expensive, and more acceptable for people than synthetic medicines. However, herbs have risks and sometimes can lead to poisoning if not used properly, and some of them are inefficient in the treatment of certain emergency cases [1].

There is a worldwide epidemic of overweight and obesity, which are usually associated with several pathologies such as diabetes mellitus, cardiovascular diseases, musculoskeletal disorders, and some types of cancer including colon, kidney, gallbladder, prostate, liver, ovarian, breast, and endometrial [2].

Diabetes is known by a long term of hyperglycemia with disturbances in the metabolism of proteins, fats, and carbohydrates, which resulted from defects in insulin action and/or insulin secretion. The main goal of any antidiabetic medicines is to reach normoglycemia to prevent microvascular and macrovascular complications [3].

Bacterial infections are considered a worldwide problem and are recognized as a threat to the life of humankind. In recent years, antibacterial and antifungal resistance has become an emergent issue in health worldwide. This resistance is mainly caused by the misuse of antibiotics [4].

According to the World Health Organization (WHO) surveys, cancer is one of the leading causes of death around the globe and responsible for about 10 million deaths in 2018. About 1 out of 6 people died from cancer which is considered the hugest cause of death, which is a considerably alarming estimate. The WHO has recognized that 1.16 trillion US dollars were spent on the prevention and treatment of cancer in 2010 alone, and that number has increased dramatically over the years [5].

*Parietaria judaica* L. (PJ) is commonly known as pellitory which belongs to the Urticaceae family and wildly growing in the northern countries of Africa, western regions of Asia, and the southern parts of Europe [6]. It is a perennial upright or spreading herbaceous plant reaching 1 m in height. The stems are greenish-brown or reddish-brown, are often much-branched, and are covered in irregularly curled hairs. The leaves have an oval shape, which is covered in irregular hairs, and they also have glossy upper surfaces. The flowers are borne in small, dense clusters in the leaf forks. They are initially greenish, but often turn reddish or reddish-brown as they mature [7].

The aqueous and lipophilic solutions prepared from PJ leaves have been broadly exploited for its medicinal value for centuries. This herb is used in traditional medicine for the treatment of kidney and bladder stones and to remove plaques deposited on the teeth. Furthermore, it is used for more thousand years as a diuretic and sedative as well as for the treatment of chronic cough, inflamed wounds, and burns [8, 9].

The current investigation is aimed at finding out more about the phytoconstituents and total tannin constituent of PJ and at investigating its antimicrobial effects against eight lethal microbial strains. Moreover, the plant antiobesity, antidiabetic, and cytotoxic characters against the HeLa cancer cell line were studied.

## 2. Material and Methods

### 2.1. Collection of the Plant Material

The leaves of PJ were gathered in November 2018 from the Jenin area of Palestine. The plant was recognized by Dr. Nidal Jaradat specialist in medicinal plants, and the voucher specimen was deposited in the Pharmacognosy Laboratory, Faculty of Medicine and Health Sciences at An-Najah National University (Pharm-PCT-1790).

The leaves were washed three times using distilled water and completely dried in the shade at room temperature. The dried parts were grounded coarsely using a mini mill machine and latterly stored in tightly sealed special containers for further use.

### 2.2. Four Solvent Exhaustive Fractionations

The dried leaves were exhaustively extracted by the fractionation method utilizing four solvents with various degrees of polarities including methanol (Loba/Chemie, India), water, acetone (Riedel/dehaen, Germany), and hexane (Alfa۔Aesar, UK). Briefly, 100 g of the dried plant leaves was taken and placed in a bottle and then extracted with 1 L of each solvent separately. Each bottle containing the plant leaves and the solvents was soaked for 72 h in a shaker device (Daihan Labtech, S. Korea) at 100 rotations per minute at 25°C. Each solvent was filtered utilizing a suction filtration. Then, all the organic fractions were dried using an incubator device (Esco, 2012-74317, Singapore) at 25°C until completely dried. A freeze dryer (Mell rock, China) was used in the drying of water fraction. Each obtained dried fraction was stored in the refrigerator at a temperature of 2-8°C for later use (5).

### 2.3. Phytochemical Screening

It is well known that plants produce many organic chemical compounds that are biologically active, not just in themselves, but also in other organisms. Some of these chemicals enhance the plants' survival. Preliminary phytochemical analysis of secondary and primary metabolic compounds such as cardiac glycosides, flavonoids, saponins, proteins, phenols, carbohydrates, and tannins was carried out according to the standard phytochemical methods [10, 11].

### 2.4. Procyanidin Determination

For the determination of total procyanidin content, the Sun et al. protocol was followed with minor modification [12]. Catechin (Sigma, USA) was used as the reference compound to construct the calibration curve for the required calculations in which a 100 *μ*g/ml stock methanolic solution was prepared; then, serial dilutions were obtained (10, 30, 50, 70, and 100 *μ*g/ml). Then, a 4% methanolic vanillin (Alfa۔Aesar, UK) solution was freshly prepared, and a 100 *μ*g/mL stock solution was made from the methanolic plant fraction using methanol as the solvent. For the working solution, each test tube contained 0.5 ml of the PJ plant methanolic fraction mixed with 3 ml of vanillin solution and 1.5 ml of concentrated HCl (SDFCL, India). The obtained mixture was allowed to stand for 15 min, and then, the absorption was measured at 500 nm against methanolic vanillin as a blank. All the working samples were analyzed in triplicate. The total procyanidin content in the plant fraction is expressed as Catechin equivalents (mg of CAE/g of the dry plant fraction).

### 2.5. Porcine Pancreatic Lipase Inhibitory Assay

The porcine pancreatic lipase inhibitory method was followed in this study according to the protocol of Bustanji et al., with minor modifications [13]. Briefly, a stock solution of 500 *μ*g/ml from each plant fraction was dissolved in 10% DMSO which was used to prepare five different solutions with the following concentrations: 50, 100, 200, 300, and 400 *μ*g/ml. One mg/ml stock solution of pancreatic lipase enzyme was obtained which is an enzyme that breaks down triglycerides into free fatty acids and glycerol. It is present in pancreatic secretions and is responsible for fat digestion and plays a crucial role in lipid transport. This enzyme was freshly prepared in the tris-HCl buffer before use. The substrate used for this study, *p*-nitrophenyl butyrate (PNPB), was prepared by dissolving 20.9 mg in 2 ml of acetonitrile. For each working test tube, 0.1 ml of porcine pancreatic lipase (1 mg/ml) was mixed with 0.2 ml of each diluted solution series for each plant fraction. The resulting mixture was then brought to a total volume of 1 ml, by adding a Tris-HCl solution and incubated at 37°C for 15 min. Following the incubation period, 0.1 ml of PNPB solution was added to each test tube. The mixture was incubated for 30 min at 37°C. Antilipase activity of PJ plant four solvent fractions was determined by measuring the hydrolysis of the PNPB compound into *p*-nitrophenolate ions at 410 nm using a UV-Visible spectrophotometer. The same procedure was repeated for Orlistat, which was used as a standard reference compound. The equation used in this analytical study is shown below:

(1) $$\begin{array}{c} \%\text{lipase inhibition}=\frac{\left(A_{B}-A_{p}\right)}{A_{B}}\times 100\%, \end{array}$$

*A*_*B*_ is the recorded absorbance of the blank solution and *A*_*p*_ is the recorded absorbance of the (PJ) sample solution.

### 2.6. *α*-Amylase Inhibitory Activity

The *α*-amylase inhibitory activity of each extract fraction was carried out according to the standard method, with minor modifications [14]. Each plant fraction was dissolved in 3 ml of 10% DMSO and then further dissolved in buffer (0.02 M of Na_2_HPO_4_/NaH_2_PO_4_, 0.006 M NaCl, at pH 6.9) to give concentrations of 1000 *μ*g/ml, from which the following dilutions were prepared: 10, 50, 70, 100, and 500 *μ*g/ml. The porcine pancreatic *α*-amylase enzyme solution was freshly prepared at a concentration of 2 units/ml in 10% DMSO.

For working solutions, a volume of 0.2 ml of enzyme solution was mixed with 0.2 ml of each (PJ) fraction and was incubated for 10 min at 30°C. After the incubation period, 0.2 ml of a freshly prepared 1% starch aqueous solution was added to each working solution, followed by an incubation period of at least 3 min. The reaction was quenched by the addition of 0.2 ml dinitrosalicylic acid (DNSA) yellow color reagent. Each working solution was then diluted with 5 ml of distilled water and then boiled for 10 min in a water bath at 90°C. The mixture was cooled to room temperature, and the absorbance was taken at 540 nm. The blank was prepared following the same steps above, but the plant fraction was replaced with 0.2 ml of the previously described buffer. Acarbose was used as the standard reference following the same steps used for plant fractions.

The *α*-amylase inhibitory activity was calculated using the following equation:

(2) $$\begin{array}{c} \%\text{of }\alpha ‐\text{amylase inhibition}=\frac{\left(A_{B}–A_{p}\right)}{A_{B}}\times 100\%, \end{array}$$

where *A*_*B*_ is the absorbance of blank and *A*_*p*_ is the absorbance of (PJ) sample.

### 2.7. Antimicrobial Activity

The antibacterial effect was determined using seven strains of bacteria which were brought from the American Type Culture Collection (ATCC): *Pseudomonas aeruginosa* (ATCC 9027), *Escherichia coli* (ATCC 25922), *Klebsiella pneumonia*, (ATCC 13883), *Proteus vulgaris* (ATCC 8427), *Enterococcus faecium* (ATCC 700221), and *Staphylococcus aureus* (ATCC 25923) as well as against the growth of a diagnostically confirmed Methicillin-Resistant *Staphylococcus aureus* (MRSA). The antifungal activity of (PJ) samples was evaluated against the growth of *Candida albicans* (ATCC 90028). However, the antimicrobial activity of (PJ) four fractions used in this study was estimated using the broth microdilution method (7, 8).

Each PJ fraction was dissolved in 100% DMSO (dimethyl sulfoxide) (Riedeldehan, Germany) at a concentration of 100 mg/ml for hexane, methanol, and water fractions and 50 mg/ml for acetone fraction. The produced solution was filter-sterilized and then was serially microdiluted (2 folds) 11 times in sterile nutrient broth (Himedia, India). The dilution processes were performed under aseptic conditions in 96-well plates (Greiner bio-one, North America). In the microwells that were assigned to evaluate the antibacterial activities of the PJ leaf fractions, microwell number 11 contained plant free nutrient broth, which was used as a positive control for microbial growth. On the other hand, microwell number 12 contained plant-free nutrient broth that was left uninoculated with any of the test microbes. This well was used as a negative control for microbial growth. Microwell numbers 1–11 were inoculated aseptically with the test microbes. Each plant fraction was made in duplicate. All the inoculated plates were incubated at 35°C. The incubation period lasted for about 18 h for those plates inoculated with the test bacterial strains and for about 48 h for those plates inoculated with *Candida albicans*. The lowest concentration of PJ at which no visible microbial growth in that microwell was observed and considered the minimal inhibitory concentration (MIC) of the examined PJ plant four fractions (8).

### 2.8. Cell Culture and Cytotoxicity Assay

HeLa cervical adenocarcinoma cells were cultured in RPMI-1640 media, which was supplemented with 10% fetal bovine serum, 1% Penicillin/Streptomycin antibiotics, and 1% l-glutamine. Cells were grown in a humidified atmosphere with 5% CO_2_ at 37°C. Cells were seeded at 2.6 × 10^4^ cells/well in a 96-well plate. After 48 h, cells were incubated with various concentrations of the tested compounds for 24 h. Cell viability was assessed by CellTilter 96® Aqueous One Solution Cell Proliferation (MTS) Assay according to the manufacturer's instructions (Promega Corporation, Madison, WI). Briefly, at the end of the treatment, 20 *μ*l of MTS solution per 100 *μ*l of media was added to each well and incubated at 37°C for 2 h. Absorbance was measured at 490 nm.

### 2.9. Statistical Analysis

The conducted tests were determined in triplicate for the four fractions of the PJ plant. The results were expressed as the means ± standard deviation (SD). Data were compared using unpaired *t*-tests. The statistical significance was considered when the *p* value was <0.05. Statistical significance is expressed in terms of ^∗^ when the *p* value < 0.05, ^∗∗^ when the *p* value ≤ 0.001, and ^∗∗∗^ when the *p* value ≤ 0.0001.

## 3. Results

### 3.1. Phytochemical Screening

The conducted phytochemical analysis revealed the presence of tannins, saponins, and carbohydrates, in the PJ, while the flavonoids, phenols, amino acids, and cardiac glycosides were absent (Table 1).

### 3.2. Procyanidin Content

**Figure 1:**
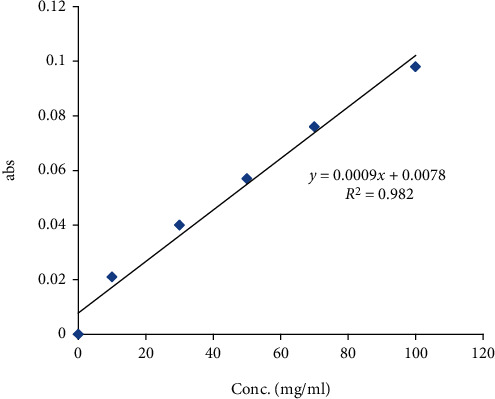
Standard calibration curve of Catechin.

According to the standard calibration curve of Catechin, as shown in Figure 1, the equation *y* = 0.0009*x* + 0.0078, *R*_2_ = 0.982 was used to estimate the total procyanidin content in the PJ plant methanolic fractions, where *y* is the absorbance at 500 nm and *x* is the total tannin content in the plant fraction. The results showed that the total procyanidin content in PJ methanolic fraction was 18.55 ± 0.55 mg of CAE/g.

### 3.3. *α*-Amylase Inhibitory Activity

Inhibition of *α*-amylase by the four different PJ fractions was detected by the previous experimental protocol and compared to Acarbose, a strong *α*-amylase inhibitory agent. The *α*-amylase inhibitory activity and IC_50_ values for the four fractions and Acarbose results are shown in Table 2. The following equations were used to calculate the IC_50_ values: for methanol fraction, *y* = 32.294*x* + 11.113; for hexane fraction, *y* = 28.015*x* + 5.4246; for acetone fraction, *y* = 18.037*x* + 2.1928; for aqueous fraction, *y* = 27.902*x* + 11.119; and for acarbose, *y* = 26.498*x* + 11.427, where *y* is the enzyme % inhibition and *x* is the logarithm concentration.

The results showed that the methanolic PJ fraction has a powerful *α*-amylase inhibitory activity even more potent than Acarbose with IC_50_ values of 15.84 ± 2.25 and 28.18 ± 1.22 *μ*g/ml, respectively.

### 3.4. Lipase Inhibitory Activity

**Figure 2:**
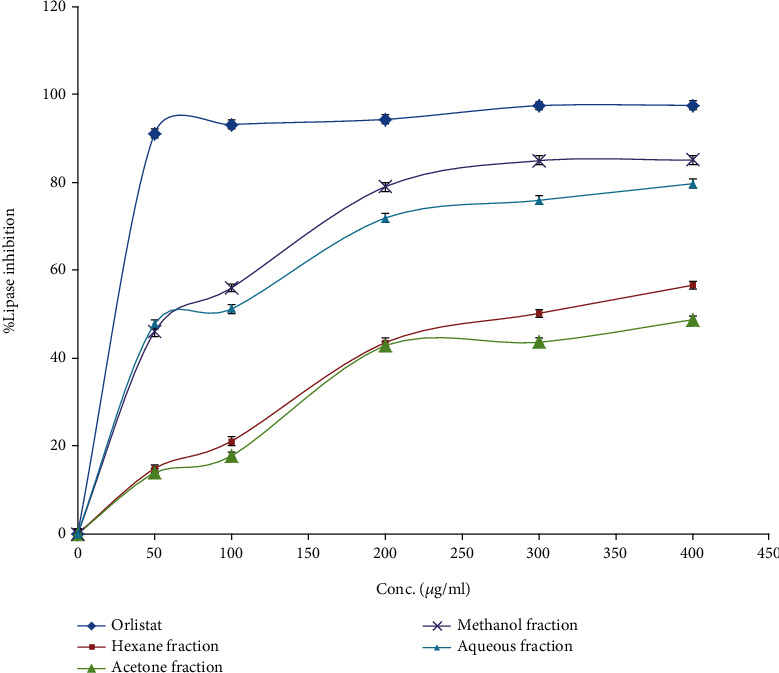
The lipase inhibition percentage of the different (PJ) four fractions compared to Orlistat.

The hydrolysis of *p*-nitrophenyl butyrate to *p*-nitrophenol was used to measure the influence of the four PJ fractions on the porcine pancreatic lipase enzyme. The results of the lipase enzyme inhibitory activity and the lipase inhibition IC_50_ values for the four fractions and Orlistat are shown in Figure 2. The following equations were used to calculate the IC_50_ values: for Orlistat, *y* = 38.639*x* + 7.6676; for methanol fraction, *y* = 33.614*x* − 3.5112; for acetone fraction, *y* = 18.255*x* − 5.8919; for hexane fraction, *y* = 20.581*x* − 6.9327; and for the aqueous fraction, *y* = 30.588*x* − 2.0254, where *y* is the enzyme % inhibition and *x* is the logarithm concentrations. The obtained results indicate that the methanol fraction has the highest antilipase activity with an IC_50_ value of 38.9 ± 0.29 *μ*g/ml, followed by the aqueous and hexane fractions with IC_50_ doses of 50.11 ± 0.57 and 588 ± 0.66 *μ*g/ml, respectively, while the acetone fraction was inactive. However, Orlistat the reference antiobesity drug has an IC_50_ value of 12.3 ± 0.35 *μ*g/ml.

### 3.5. Antimicrobial Activity

In the current investigation, the broth microdilution method was utilized to evaluate the antimicrobial effect of PJ four fractions. In fact, this method is the most popular worldwide, because of its accuracy and clear results. In this method, wells were filled with a broth containing different concentrations of the PJ plant different fractions to assess their antimicrobial effects.  Table 3 illustrates that the PJ methanol, hexane, and acetone fractions have various degrees of antibacterial and antifungal activities against the tested strains while the aqueous fraction did not show antibacterial and antifungal effects.

### 3.6. Cytotoxic Effect

**Figure 3:**
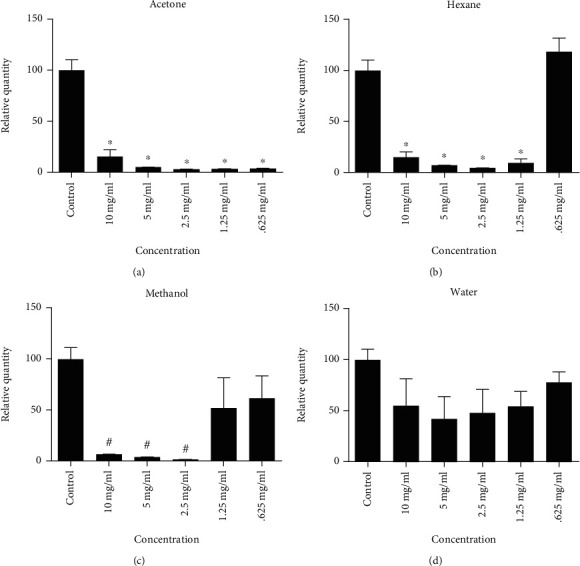
The cytotoxic effect of (PJ) four solvent fractions on HeLa cells which were treated with different concentrations of (a) acetone, (b) hexane, (c) methanol, and (d) water fractions incubated for 24 h. Results were depicted as relative quantities (RQs) compared to the control (with only media). ^∗^*P* < 0.0001 and ^#^*P* < 0.01. Error bars represent ±SD.

In the present study, the antiproliferative potential of PJ solvent fractions, namely, aqueous, methanol, acetone, and hexane, was investigated on HeLa cervical adenocarcinoma. HeLa cells were exposed to increasing concentrations of each tested plant sample (0.625, 1.25, 2.5, 5, and 10 mg/ml) for 24 h, and the cell viability was quantified using an MTS assay. Treating HeLa cells with 0.625, 1.25, 2.5, 5, and 10 mg/ml of PJ acetone fraction (Figure 3(a)), cell viability significantly decreased (*p* value < 0.0001) by approximately 90%. As demonstrated in Figure 3(b), hexane fraction decreased cell viability significantly (*p* value < 0.0001) by approximately 90% at all tested concentrations except the lowest concentration (0.625 mg/ml). Treatment of HeLa cells with 2.5, 5, and 10 mg/ml of methanol fraction decreased cell viability significantly (*p* value < 0.01) by approximately 90%, while the rest of the concentrations had no significant effect (Figure 3(c)). As shown in Figure 3(d), water extract had no significant effect at all tested concentrations.

## 4. Discussion

Anthocyanins and other phenolic compounds isolated from medicinal plants have recently raised considerable interest and have received increasingly more attention due to their bioactive effects [15, 16]. These components are classified as secondary plant metabolites and usually exert antimicrobial, anticancer, anti-inflammatory, and antiviral effects along with their high antioxidant activity [17, 18].

In fact, polyphenolic compounds have been reported to possess various therapeutic actions, including anticancer, antidiabetic, and antiobesity. Many plant products and plant extracts have shown significant antiobesity and antidiabetic activities, which may be an important property of medicinal plants associated with the treatment of several illnesses including atherosclerosis, diabetes, and obesity [19].

The phytochemical screening of PJ showed the presence of carbohydrates, proteins, saponins, and tannins and revealed that the methanolic fraction of this plant contains 18.55 ± 0.55 mg of procyanidin.

Procyanidin and anthocyanin are polyphenolic groups of natural products and play an essential role in the human diet, even though many medical practitioners, epidemiologists, nutritionists, and food scientists have related a decrease in obesity and its related diseases such as diabetes to the high ingestion of plants containing these potent molecules [20]. The obtained results from the current study showed that the methanolic fraction containing procyanidin has a powerful action against the *α*-amylase metabolic enzyme. Actually, this enzyme is responsible for the digestions of the food containing complex polysaccharides to convert them to monosaccharide units. The obtained *α*-amylase inhibitory activity outcomes were compared with a commercial antidiabetic drug Acarbose. The methanolic, hexane, aqueous, and acetone fractions of PJ plant have *α*-amylase inhibitory activity IC_50_ values of 15.84 ± 2.25, 38.9 ± 2.38, 158.4 ± 0.9, and 28.18 ± 1.22 *μ*g/ml, respectively, while Acarbose has an IC_50_ value of 28.18 ± 1.22 *μ*g/ml. A study was conducted in Jordan by Hamdan and Afifi on the *α*-amylase inhibitory activity of *Paritaria diffusa* leaves who found that the hydromethanol extract was inactive [21].

Moreover, the methanol PJ fraction has a potential antilipase effect followed by the aqueous and hexane fractions with IC_50_ values of 38.9 ± 0.29, 50.11 ± 0.57, and 588 ± 0.66 *μ*g/ml, respectively, while the commercial antiobesity drug Orlistat was used as a reference compound and has antilipase activity with an IC_50_ value of 12.3 ± 0.35 *μ*g/ml.

To the best of our knowledge, no previous studies were conducted on the effect of any of *Parietaria* plant genus on lipase enzymes inhibitory activity.

Nevertheless, the folkloric remedies have always been known as a rich source of phytochemicals which are important for the discovery of potent new medications especially these compounds that are working on communicable diseases [22].

Antimicrobial activity evaluation of PJ solvent fractions showed that the methanolic fraction exhibited the growth only of *S. aureus* and *P. aeruginosa* with MIC values of 25 and 12.5 mg/ml, respectively, while the acetone fraction inhibited the growth of all the tested bacterial strains including *S. aureus*, *E. coli*, *P. vulgaris*, *E. faecium*, *P. aeruginosa*, and MRSA. The highest inhibition was against the growth of *S. aureus* with a MIC dose of 1.56 mg/ml. Moreover, the hexane fraction inhibited the growth of all the screened bacterial strains. Unfortunately, the aqueous fraction of PJ did not inhibit the growth of the evaluated strains. Regarding antifungal activity, the PJ acetone and hexane fractions showed powerful anticandidal activity with MIC values of 0.195 and 0.78 mg/ml. These numbers suggest that the PJ has powerful antifungal activity against *C. albicans*.

In a study conducted by Fares et al., it was found that the aqueous and ethanolic extracts of PJ have antibacterial activity against *S. pneumoniae* with MIC doses of 3.125 and 100 mg/ml, respectively [23]. Another study showed that the ethanol and methanol extracts of PJ have antibacterial activity against multidrug-resistant *E. coli* with MIC values of 6.25 and 50 mg/ml, respectively [24].

To the best of our knowledge, no previous studies were conducted on the antifungal activity of PJ and it is interesting to show that its acetone fraction has powerful anticandidal activity with a MIC value of 0.195 mg/ml.

Pollinosis from PJ plant is known to cause allergy [25]; this may explain the little interest and consequently scarcity of studies investigating the biological activities of this plant including cytotoxicity. A recent study found that the ethanol extract of PJ has a cytotoxic effect on prostate cancer cell lines, namely, PC-3, DU145, and HDF cell lines and the IC_50_ values were >300 *μ*g/ml [26]. This is a little stronger than what we observed. However, according to the reported classification [27], acetone extract has a weak toxic effect, while the rest of the extracts have no cytotoxic effect. This suggests that all extracts of PJ are safe to use.

The above results illustrate that the methanol, hexane, and acetone fractions of PJ could be a possible source for an effective drug treating cancer and bacterial and fungal infections. However, it is important to isolate bioactive compounds from PJ and assess their biological activities in vivo.

### 4.1. Significance of the Study

Acarbose is an antidiabetic drug that acts by inhibiting *α*-amylase and *α*-glucosidase enzymes but with deleterious side effects [28]. The lipase inhibitor Orlistat is currently the sole antiobesity agent available in many countries [29]. However, gastrointestinal side effects are common and may limit the use of Orlistat [30]. Antibiotics are frontline therapy against microbial infectious diseases and many antibiotics are known to cause several side effects in humans [31]. Although the effectiveness of cancer treatments has improved over time, adverse effects persist with each treatment [32]. The significance of this work came from the ability of PJ to inhibit *α*-amylase and *α*-glucosidase and inhibit gastric and pancreatic lipase, in addition to the antibacterial, antifungal, and antiproliferation effects of PJ. All this happens naturally by PJ contents without any reported side effects.

## 5. Conclusion

The PJ methanolic fraction has high contents of procyanidin and has a potential *α*-amylase and lipase inhibitory activity compared to Acarbose and Orlistat drugs. However, the PJ acetone, hexane, and methanol fractions have broad-spectrum antibacterial agents while the acetone fraction revealed potent antifungal activity against *Candida albicans*. At low concentrations, hexane and acetone fractions had potent cytotoxic and antiproliferative activity against HeLa cervical adenocarcinoma cancer cells. Moreover, PJ acetone, hexane, and methanol fractions can serve as an effective source of natural products to develop new antiobesity, anticancer, antimicrobial, and hypoglycemic agents.

## Figures and Tables

**Table 1:** Phytochemical screening results of (PJ) plant four solvent fractions.

Aqueous fraction	Hexane fraction	Acetone fraction	Methanol fraction	Phytochemical classes
—	—	—	—	Amino acids and protein
+++	—	—	+	Carbohydrate
—	—	—	++	Tannin
—	—	—	—	Flavonoid
—	—	—	—	Phenol
++	—	—	—	Saponin
—	—	—	—	Cardiac glycoside

—: no content; +: content; ++: high content.

**Table 2:** *α*-Amylase inhibitory activity and IC_50_ values **(***μ*g/ml) of different (PJ) fractions compared to Acarbose (±SD).

Conc.	Acarbose	Hexane fraction	Acetone fraction	Methanol fraction	Aqueous fraction
0	0 ± 0	0 ± 0	0 ± 0	0 ± 0	0 ± 0
10	53.22 ± 1.2	43.35 ± 0.82	28.32 ± 4.08	59.53 ± 1.63	51.44 ± 0.81
50	54.91 ± 0.58	47.69 ± 0.41	29.19 ± 4.49	59.82 ± 1.13	59.54 ± 0.82
70	66.1 ± 1.34	57.05 ± 0.23	32.08 ± 4.49	75.14 ± 1.63	68.78 ± 0.81
100	66.1 ± 1.62	65.6 ± 9.2	36.12 ± 1.22	82.37 ± 1.23	70.23 ± 0.41
500	72.54 ± 1.37	77.16 ± 1.22	53.75 ± 1.63	87.57 ± 0.41	73.98 ± 1.63
IC_50_	28.18 ± 1.22	38.9 ± 2.38^∗^	446 ± 3.18^∗∗^	15.84 ± 2.25^∗∗∗^	158.4 ± 0.9^∗∗^

^∗^ *p* value < 0.05, ^∗∗^*p* value ≤ 0.001, and ^∗∗∗^*p* value ≤ 0.0001.

**Table 3:** The antimicrobial activity MIC values (mg/ml) of (PJ) four solvent fractions.

(PJ) fractions	*S. aureus*	*E. coli*	*K. pneumonia*	*P. vulgaris*	*E. faecium*	*P. aeruginosa*	MRSA	*C. albicans*
Methanol	25	R	R	R	R	12.5	R	R
Acetone	1.56	6.125	R	3.125	6.125	6.125	3.125	0.195
Hexane	6.125	12.5	12.5	6.125	6.125	6.125	6.125	0.78
Water	R	R	R	R	R	R	R	R

R: resistant.

## Data Availability

No data were used to support this study.
